# Towards a unified regional surveillance system: the Gulf CDC’s first initiatives

**DOI:** 10.3389/fpubh.2024.1478679

**Published:** 2025-01-03

**Authors:** Sami Al Mudarra, Lamis Alghamdi, Ahmed AlHatlan, Andrew Amato Gauci, Aleksandra Polkowska Kramek, Alaa Alqurashi, Nouf Alzuhayri, Sadeem AlShiban, Bushra Alghamdi, Pasi Penttinen

**Affiliations:** Gulf Center for Disease Control and Prevention (CDC), Riyadh, Saudi Arabia

**Keywords:** Gulf region, surveillance, Gulf Centre for Disease Prevention and Control, public health, disease indicators, health information

## Abstract

In January 2021, the Gulf Health Council (GHC), established the Gulf Centre for Disease Prevention and Control (Gulf CDC) in Riyadh, marking a pivotal step in harmonizing health strategies, enhancing knowledge generation, and promoting evidence-based approaches to both communicable (CD) and non-communicable diseases (NCD). The Gulf CDC’s mission includes consolidating the region’s health information systems, crucial for monitoring disease burden and shaping effective public health policies. An initial assessment of public health surveillance systems across the Gulf Cooperation Council (GCC) member states was conducted by the Gulf CDC. This revealed strong national surveillance coverage of CDs but identified areas for improvement, particularly in data quality and representativeness. These findings informed the development of the Gulf CDC’s health information strategy and confirmed the need for a regional surveillance system. Prior to the introduction of this system, senior experts in this field from all GCC member states were surveyed and a consensus process launched to agree on the first steps. This led to the strategic selection of a small number of priority communicable and non-communicable diseases for the pilot phase of the system. The final agreed list of diseases and conditions for the pilot are the CDs: acute respiratory infection (ARI), endemic dengue, brucellosis, measles and pulmonary tuberculosis together with the NCDs: cancer (registry) and road traffic injuries. The initiative will showcase the potential benefits of regional collaboration to improve health outcomes and will ultimately also contribute to global health security efforts.

## Introduction

The Gulf Health Council (GHC), established nearly five decades ago by the Gulf Cooperation Council (GCC), has played a pivotal role in advancing public health across Gulf states (1). In a landmark move in January 2021, the GHC launched the Gulf Centre for Disease Prevention and Control (Gulf CDC), headquartered in Riyadh, to drive harmonization, knowledge generation, and promote evidence-based strategies in the prevention and mitigation of communicable (CD) and non-communicable diseases (NCD). The Gulf CDC is also charged with tackling public health emergencies and fostering healthier communities across the Gulf region (2).

Central to the Gulf CDC’s mission is the consolidation of the region’s health information systems, so important for monitoring disease burden, understanding health determinants, and developing effective public health policies. As a first step, the Gulf CDC assessed the GCC member states’ public health surveillance systems, utilizing the 2001 Guidelines for Evaluating Surveillance Systems from the US Centres for Disease Control and Prevention (CDC) (3). This assessment evaluated these systems across several indicators including usefulness, simplicity, flexibility, and data quality. Overall, the evaluation revealed strong surveillance coverage of communicable diseases across the six member states. It also identified areas for improvement, particularly in data quality and representativeness (4). These insights were pivotal in shaping the Gulf CDC’s health information strategy, aimed at enhancing the quality and comparability of national and regional epidemiological data.

## Building consensus on regional surveillance indicators

A critical next step is agreement on the priority health and disease indicators for piloting the new Gulf CDC’s regional surveillance system. This process involved two surveys conducted among eighteen senior surveillance and data experts from all the GCC member states, followed by a consensus workshop in June 2023. The initial survey collected the opinions on a wide range of issues including the potential lists of CDs and NCDs to be included in the surveillance system.

Based on the findings of the first survey, the second survey produced a potential shortlist suitable for piloting the system, as well as their reporting frequency. The CDs proposed were COVID-19, seasonal influenza, dengue, malaria, measles, tuberculosis, and antimicrobial resistance (AMR). For NCDs, cancer and road traffic accidents were proposed due to the existing infrastructures, such as cancer registries, that could facilitate the piloting of the system. The survey also proposed several reporting frequencies—weekly, monthly, quarterly, and annually—depending on the disease and data needs.

The face-to-face workshop enabled real-time discussion and consensus-building between the national experts on the final selection for inclusion in the pilot. The dialogue on each aspect followed the results produced in real time by an audience response system where the experts could anonymously share their choices. Through these discussions, several strategic adjustments were made to the draft list identified earlier. Notably, AMR was excluded from the pilot phase due to the complexities associated with its surveillance, with brucellosis included instead, recognizing that the lower incidence will allow for a more manageable data load. Additionally, it was agreed that COVID-19 and seasonal influenza be reported under the broader category of Acute Respiratory Infections (ARI), which would also cover Middle East Respiratory Syndrome (MERS) and potentially Respiratory Syncytial Virus (RSV) infection.

Cancer was confirmed as the most suitable non-communicable disease for the pilot phase, owing to the well-established cancer registries across the member states, with annual reporting deemed adequate. Road traffic accidents were also accepted, despite some concern of the challenges of standardizing data across multiple sources. Regarding the frequency of reporting, the experts appropriately recommended that these should align with existing international reporting systems such as for WHO to minimize the burden of reporting (5). The decision of diseases and conditions for the pilot phase of the surveillance system is summarised in Table 1.

**Table 1:** Communicable and non-communicable diseases reportable to the Gulf CDC (2023–2025), phase one.

Reported disease/events	Frequency of reporting	Case definition
Cancer registry data	Annually	Following IARC guidelines (10)
Road traffic injuries data	Annually	To be defined
Acute Respiratory Infection (ARI)	Weekly	Aligned as far as possible with those used by WHO
Endemic dengue	Weekly	
Brucellosis	Annually	
Measles	Monthly	
Pulmonary tuberculosis	Annually	

## Discussion

The Gulf CDC’s initiative to create a regional surveillance system is a groundbreaking step forward in public health collaboration within the GCC. Aside from the valuable data generated, the system should foster greater collaboration and trust among the important stakeholders, such as health care providers, laboratories, public health agencies, and communities (6, 7).

It is recognised that the way ahead is expected to present several challenges. During the consensus meeting, the experts raised data sharing, data quality, representativeness, timeliness, transparency, and trust in the system as their main concerns. Currently data standardisation is also an issue, and this needs to be tackled by developing a unified solid and reliable user-friendly platform for standardised reporting and data sharing. Such a platform/reporting system must take into consideration the limitations of the current national systems and their resources and be aligned with the WHO standards and data requirements, wherever possible. Finally, to reduce the strain on already overburdened national surveillance teams, the burden and cost of data collection, reporting, and analysis must be minimized by preventing duplication, inconsistency, and inefficiency.

Over all however this initiative not only has the potential to significantly improve health outcomes for millions in the Gulf region but by focusing on harmonizing surveillance efforts, leveraging advanced technologies, and enhancing regional integration, it will offer a blueprint for other global health agencies seeking to improve their disease monitoring and response mechanisms.

Moreover, the Gulf CDC is mindful of the importance of aligning regional surveillance systems with international standards, such as those set by the World Health Organization (WHO) (5), to ensure consistency, reduce reporting burdens, and strengthen global health security. As the work progresses beyond the pilot phase, the lessons learned, and the data generated is expected to contribute to the Global Health Security Agenda (8) and improve the region’s support to the International Heath Regulations (9).

By addressing the challenges related to data quality, timeliness, and transparency, and by harnessing the latest technological advancements, the Gulf CDC is positioned to create an effective and sustainable surveillance system. This initiative will not only strengthen the public health infrastructure of the GCC member states but also promotes the region as a leader in global health security.
